# Coagulation-related genes for thyroid cancer prognosis, immune infltration, staging, and drug sensitivity

**DOI:** 10.3389/fimmu.2024.1462755

**Published:** 2024-10-21

**Authors:** Yuxiao Sun, Yifei Zhang, Yuchuan Yang, Weihao Liu, Detao Yin

**Affiliations:** ^1^ Department of Thyroid Surgery, The First Affiliated Hospital of Zhengzhou University, Zhengzhou, China; ^2^ Department of Radiotherapy, The First Affiliated Hospital of Zhengzhou University, Zhengzhou, China; ^3^ Engineering Research Center of Multidisciplinary Diagnosis and Treatment of Thyroid Cancer of Henan Province, Zhengzhou, China; ^4^ Key Medicine Laboratory of Thyroid Cancer of Henan Province, Zhengzhou, China

**Keywords:** thyroid cancer, coagulation, prognosis signature, immune infiltration, clinical relevance, drug sensitivity

## Abstract

**Introduction:**

Thyroid cancer (THCA) is the most common endocrine tumor. Coagulation may be associated with the development of cancer, but its role in THCA patients is not yet clear.

**Methods:**

In this study, we determined the predictive value of coagulation biomarker D-dimer for THCA patient lateral lymph node metastasis (LLNM) through receiver operating characteristics (ROC) analysis and logistic regression analysis. Subsequently, this study used the TCGA database to identify coagulation-related molecular subtypes through consensus clustering analysis and compared their prognosis. We identified coagulation-related genes (CRGs) associated with prognosis in thyroid cancer through gene expression data and clinical information, and constructed a prognostic model by selecting the prognostic CRGs using LASSO regression. Patients were divided into high-risk and low-risk groups based on the median score. Subsequently, prognosis, clinical characteristics, gene mutation occurrence, immune infiltration, function, and drug sensitivity of the two groups were analyzed. We also constructed a nomogram combining the model and clinical features. Finally, the expression of the prognostic CRGs was validated by RT-qPCR.

**Results:**

D-dimers had better performance in predicting LLNM(the area under the curve was 0.656 (95% CI 0.580-0.733), with a cut-off value of 0.065 mg/l), and D-dimer>0.065mg/l was an independent predictor of LLNM. Then, we selected 8 prognostic CRGs to construct a predictive model. The prognosis of low-risk group patients was significantly better than that of high-risk group (P<0.001). The results showed significant differences in clinical characteristics, gene mutation occurrence, immune infiltration, function, and drug sensitivity between the high-risk and low-risk groups. We validated by qPCR that these 8 prognostic CRGs were overexpressed in THCA cell lines.

**Discussion:**

Overall, this study provided an in-depth exploration of the potential role of the coagulation in thyroid cancer and its clinical significance, offering a new theoretical basis and research direction for personalized therapy and prognostic evaluation.

## Introduction

Thyroid carcinoma (THCA) is the most prevalent endocrine tumor (1), and the incidence rate of THCA in China has significantly increased since 2000, with an estimated 22,000 new cases reported in 2022 (2). THCA is classified into three pathological types: differentiated thyroid cancer (DTC), medullary thyroid cancer (MTC), and anaplastic thyroid cancer (ATC) (3). Despite the generally good prognosis for most thyroid cancer patients, 10%-15% of patients experience recurrence, and 5% develop distant metastases (4). To further improve the prognosis of THCA patients, it is important to identify suitable prognostic biomarkers.

Coagulation, one of the hallmarks of tumor, could be a consequence of increasing plasma extravasation and vascular permeability which leads to extravascular coagulation, or be activated by disruption of vessels which leads to intravascular coagulation (5). A study has shown that tumor cells can release procoagulant factors, such as tissue factor, which may trigger the coagulation cascade both *in vitro* and *in vivo* (6). On the other hand, tumor coagulum, a cancer-driven network of molecular effectors favoring bleeding or thrombosis, could interact with the tumor microenvironment (TME) to orchestrate cancer inhibition or progression (7). Additionally, thyroid hormones directly regulate the transcription of genes encoding coagulation proteins in hepatocytes and endothelial cells (8). Therefore, the coagulation pathway may interact with the occurrence and development of THCA, but the role of coagulation in THCA patients remains unclear.

In this study, using The Cancer Genome Atlas (TCGA) cohort, we identified coagulation-related molecular subtypes via consensus clustering analysis and compared their prognoses. We used gene expression data and clinical information from TCGA’s THCA to identify differentially expressed Coagulation-related genes (CRGs) in THCA and analyzed their correlation with prognosis. Through LASSO regression, we further filtered out 8 prognostic CRGs to construct a prognostic model and examined the relationships between risk scores and clinicopathological features, molecular functions, pathways, outcomes, immune infiltration, and immunotherapy. Additionally, we assessed the predictive performance of coagulation indicators for LLNM.

## Methods

### Patient selection and data collection

We gathered and screened data from patients admitted to the First Affiliated Hospital of Zhengzhou University from March 1, 2024, to May 1, 2024. The inclusion criteria were: (1) first-time thyroid surgery; (2) histologically confirmed papillary thyroid cancer (PTC); (3) age >18 years. The exclusion criteria were: (1) presence of other malignancies; (2) incomplete clinical data; (3) presence of diseases related to abnormal coagulation levels (including venous thromboembolism, disseminated intravascular coagulation, etc.). Finally, 408 patients were involved in our research. Age, gender, presence of Hashimoto’s thyroiditis (HT), presence of extrathyroidal extension (ETE), multifocality of PTC, primary tumor size, and presence of lymph node metastasis (N stage, including central lymph node metastasis (CLNM) and lateral lymph node metastasis (LLNM)) were extracted from medical records. All patients underwent coagulation marker tests within two weeks before surgery. The antibody positivity often precedes clinical manifestations of thyroid dysfunction or sonographic changes in patients with HT. Studies have shown that elevated anti-TPO or anti-Tg antibodies can be present for years before the development of overt hypothyroidism or characteristic ultrasound changes, making antibody testing a valuable early diagnostic tool (9). HT was diagnosed by postoperative sectioning and examination of paraffin-embedded thyroid tissue specimens. Additionally, Serum antithyroglobulin and antithyroid peroxidase levels were measured within 30 days before surgery using the immuno-electrochemiluminescence method, and the patients were diagnosed with HT when these levels exceeded 115 IU/ml and 34 IU/ml, respectively. ETE referred to breaking through the thyroid capsule and invading adjacent soft tissues, muscles, trachea, oesophagus, nerves or blood vessels. Bilateral disease and multifocal disease were considered together for statistical analysis. Multifocality was defined as the presence of more than one lesion observed (10). Primary tumor size greater than 2 cm has been identified as an important risk factor for recurrence and lymph node metastasis in papillary thyroid carcinoma (3).

### Data download and organization

**Figure 1:**
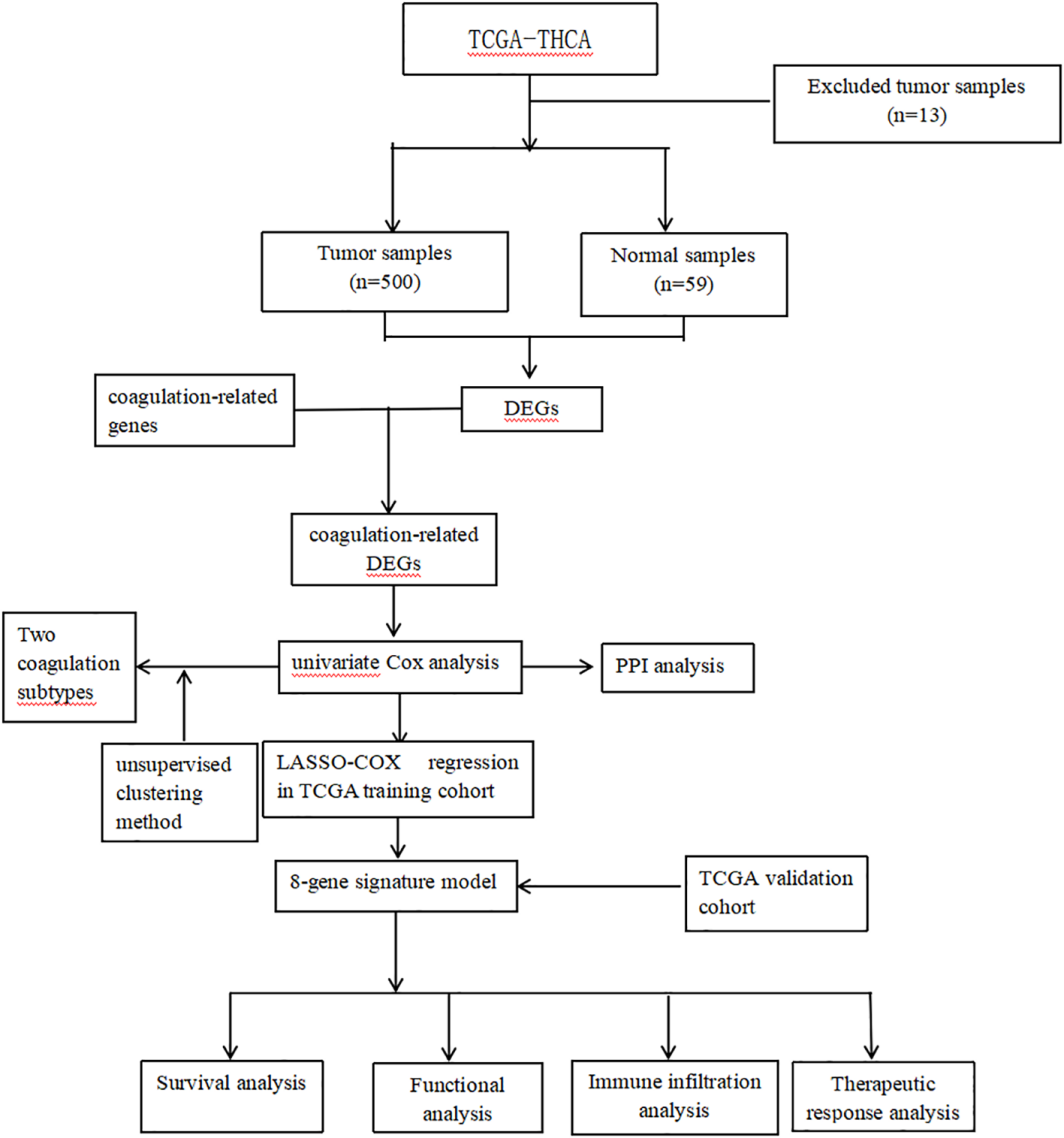
Flow chart of analysis.

The flow chart is shown in **Figure 1**. The RNA-seq data, clinical information and mutation data of THCA were acquired from the TCGA-THCA. After removing data with missing or less than 30 days of follow-up, missing outcome time occurrence status, and duplicates, the TCGA-THCA set remained 500 THCA samples. Based on the GeneCards website (https://www.genecards.org/), we retrieved 378 coagulation-related genes (CRGs, Relevance Score≥3) via searching the term “coagulation” (7, 11).

### Collection of differentially expressed CRGs

The “limma” R package was conducted to figure out differentially expressed genes (DEGs) between normal and tumor tissues (FDR < 0.05 and log2FC >=1) in TCGA-THCA sets. Then, we took the intersection of these DEGs and CRGs to obtain differentially expressed CRGs (DECRGs). To explore the prognostic value of DECRGs in THCA, univariate Cox analysis was conducted in TCGA-THCA set (p < 0.05). Te co-expression network of the prognostic DECRGs was explored by the “igraph” R package. Using the STRING (https://string-db.database, the protein-protein interaction (PPI) network org/) of proteins coded by the mitochondrial dynamic prognostic DECRGs was constructed and visualized. Finally, we validated these prognostic DECRGs using survival analyses and log-rank tests.

### Identification of coagulation subtypes and survival analysis

Based on these prognostic DECRGs, we used the “ConsensusClusterPlus” package to perform consensus clustering on THCA samples from the TCGA-THCA dataset. To determine the optimal number of clusters, we used the Consensus Cumulative Distribution Function (CDF) to depict the CDF distribution patterns at different cluster numbers (k), and by plotting the Delta area plot to visually show the rate of change in the area under the CDF curve as the number of clusters increases from k to k+1, using this as a basis to select the optimal number of clusters and further subdivide TC patients into different subtypes. Then, we used the “survminer” package to plot Kaplan-Meier survival curves to analyze the differences in progression-free interval (PFI) between different subtypes.

### Construction of prognostic model

We randomly split the samples from the whole set into a train set and a test set according to a 6:4 ratio using the R package “caret”. To further compress these prognostic DECRGs, we performed LASSO analysis using 10-fold cross-validation (12). Finally, we constructed a prognostic model using stepwise multivariate Cox regression analysis. The scores for each sample were calculated according to the following formula: Risk score = coefficient1 * gene 1 expression +…+ coefficientN * gene N expression, and the samples were classified into high or low risk groups based on the median score.

### Validation of prognostic signature

Kaplan-Meier analysis with chi-squared test was applied to estimate survival differences between risk groups in the model. The test set, and whole set were applied to validate the internal stability of the model. The time-dependent receiver operating characteristic (ROC) curves were employed to analyze the predictive performance of the model and clinical characteristics through the R package “timeROC” (13). To assess the applicability of the model to patients with diverse clinical characteristics, we compared the survival differences between different risk score groups within each subgroup. To assess whether the model was an independent predictor for predicting patient prognosis, we performed univariate and multivariate Cox analyses including risk scores and clinical characteristics. To assess the clinical relevance of the model, the study examined the relationship between groups and clinical features. To better apply the model to clinical work, we constructed a nomogram combining the model and clinical features to predict the 3-, 5-, and 7-year survival probabilities of patients.

### Enrichment analysis and somatic mutations

To explore the differences in somatic mutations between different risk groups, we performed analysis and visualization using the R package “maftools” (14). To find differences in molecular mechanisms and relevant pathways between risk groups, we recognized differentially expressed risk genes (DERGs) between risk groups (|logFC > 1| and FDR < 0.05) and conducted Gene Ontology (GO) and Kyoto Encyclopedia of Genes and Genomes (KEGG) analyses (p < 0.05) (15–18).

### Immune microenvironment and immunotherapy

Furthermore, we employed the EPIC, MCP-COUNTER, TIMER, XCELL, QUANTISEQ, CIBERSORT, and CIBERSORT-ABS algorithms to examine immune cell infiltration (19–25). Subsequently, we scored the immune function of different risk groups using single-sample gene set enrichment analysis (ssGSEA) and compared the scores using the Wilcoxon test (26, 27). We also explored the differences in immune checkpoint gene expression levels between groups using the wilcox test. To predict the response to immunotherapy in each risk group, we calculated the tumor immune dysfunction and exclusion (TIDE) scores for each group and compared them (28). Finally, we used ESTIMATE to compare TME scores (StromalScore, ImmuneScore, and EstimateScore) across different risk groups.

### Development of individualized anti-tumor treatment protocols

In this study, the calcPhenotype function in the “oncoPredict” R package was utilized to predict drug sensitivity in the Genomics of Drug Sensitivity in Cancer (GDSC) database (29).

### Reverse transcription−qPCR (RT−qPCR)

Using qPCR technology to detect the differential expression of genes used to construct a prognostic model in human papillary thyroid carcinoma cell lines (IHH4, KTC-1, TPC-1) and normal thyroid cell lines (Nthy ori-3-1). First, TRIzol reagent (Invitrogen, USA) was used to extract total RNA from the cells. Subsequently, the extracted RNA was reverse-transcribed into cDNA using the Prime Script RT reagent kit (Takara, Japan). After obtaining the cDNA template, quantitative PCR was carried out according to the instructions of the SYBR Green Quantitative PCR Detection Kit (Takara, Japan). All the reactions were repeated for at least 3 times. GAPDH was used as an internal control gene, and the 2−△△Ct method was used for the relative quantification of the target genes. The primer sequences used in this study were: AZU1, Forward: 5′-AGAACCTGAACGACCTGATGC-3′ and Reverse: 5′-CCTGGGAAAACGGGAGAGA-3′; COL3A1, Forward: 5′-TTGCTGTGGTGGTGTTGGAG-3′ and Reverse: 5′-TTCTAGCGGGGTTTTTACGA-3′; CP, Forward: 5′-AGGAGATTCGGTCGTGTGGT-3′and Reverse: 5′-TTGAGGGAAGAGGTTTGCTG-3′; CSF2, Forward: 5′-AGAGACACTGCTGCTGAGATG-3′and Reverse: 5′-CAGGAAGTTTCCGGGGTT-3′;F12, Forward: 5′-AGGACCAGCGATGGGGATA-3′ and Reverse: 5′-TGTGGAAAAACCGGAGAAGC-3′; GNA14, Forward: 5′-TGTTACGACAGGAGGAGGGA-3′ and Reverse: 5′-CGAAGCACATCTTGTTGGGT-3′; IL1RN, 5′-AACAGAAAGCAGGACAAGCG-3′ and Reverse: 5′-CCTTCGTCAGGCATATTGGT-3′; SERPIND1, Forward: 5′-ATGGGTATGATTTCCTTAGGTCTG-3′ and Reverse: 5′-GGAAGAGATTATGAATGGTCGTG-3′; GAPDH, Forward: 5′-GGCAAATTCCATGGCACCG -3′ and Reverse: 5′-TCGCCCCACTTGATTTTGGA-3′.

### Statistical analysis

All data analyses were completed using R software (version 4.3.9) and GraphPad Prism (version 8.0.2). ROC curves were applied to evaluate the area under the curve (AUC) for each coagulation index. We used univariate and multivariate logistic regression analyses to determine the association between clinical characteristics and LLNM. Spearman correlation analysis was used to evaluate the correlation between two continuous variables. The Wilcoxon rank-sum test or Student’s T-test was used for the statistical analysis of two groups of continuous variables. Kaplan-Meier survival curves and log-rank tests were used to assess differences in DFS between different groups. P values less than 0.05 were considered to indicate statistical significance. ns indicates P>0.5, * indicates P<0.05, ** indicates P<0.01, *** indicates P<0.001.

## Results

### Predictive performance of coagulation indexes

**Figure 2:**
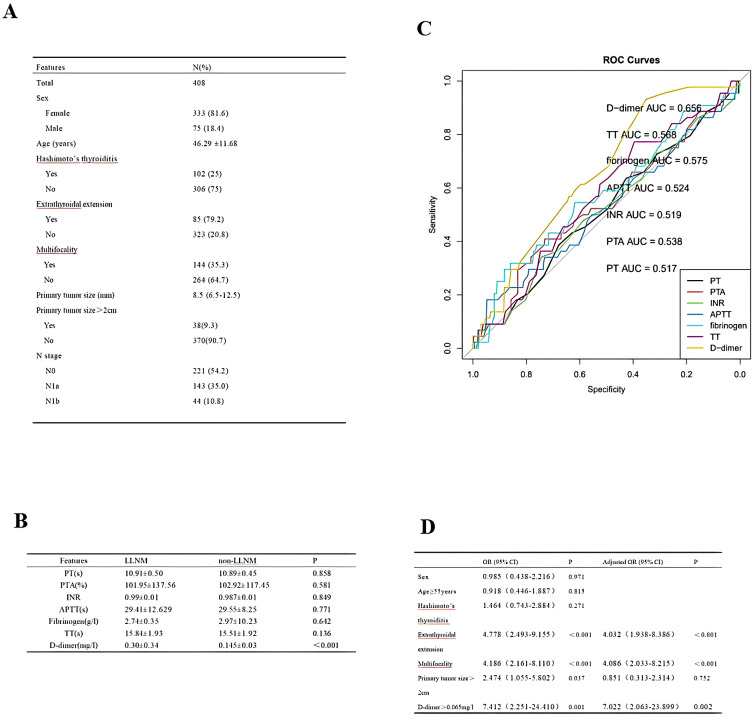
**(A)** Cohort Demographic and Clinical Characteristics. **(B)** The mean of coagulation profiles between LLNM and non-LLNM patients. **(C)** ROC curves of preoperative PT, PTA, INR, APTT, fibrinogen, TT, and D-dimer to predict LLNM in PTC patients. **(D)** Analysis of factors influencing LLNM in PTC patients.

We collected 408 cases of adult PTC, and their clinicopathological characteristics are shown in **Figure 2A**. The mean of coagulation profiles between LLNM and non-LLNM patients was compared in **Figure 2B**. The ROC curves revealed that D-dimer had superior predictive performance compared to prothrombin time (PT), prothrombin activity (PTA), international normalized ratio (INR), activated partial thromboplastin time (APTT), fibrinogen, and thrombin time (TT). The area under the curve (AUC) was 0.656 (95% CI 0.580-0.733), with a cut-off value of 0.065 mg/l (**Figure 2C**). The cut-off value for PT was 11.15s; for PTA, it was 130%; for INR, the cut-off value was 1.025; for APTT, it was 35.8s; for fibrinogen, the cut-off value was 3.175g/l; and for TT, it was 14.95s. Subsequently, through univariate and multivariate analyses, we identified the factors affecting LLNM, including extrathyroidal extension, multifocality, and D-dimer>0.065mg/l (**Figure 2D**).

### Dysregulated CRGs in THCA and their prognosis

**Figure 3:**
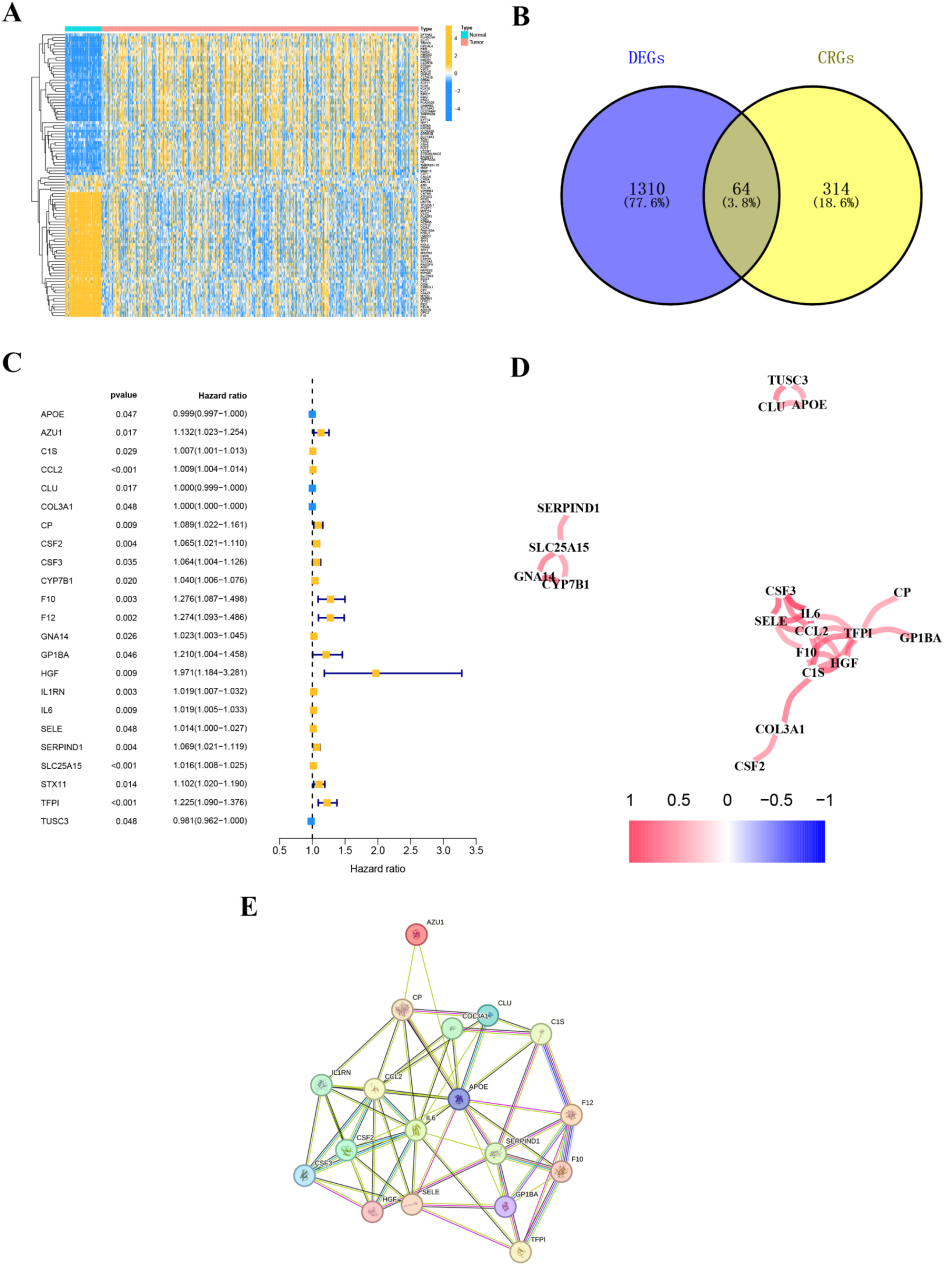
Coagulation-related differentially expressed genes. **(A)** The heatmap diagram for differential gene expression between THCA and normal tissues. **(B)** The Venn plot displaying the overlap of differentially expressed coagulation-related genes (DE-CRGs). **(C)** Univariate Cox regression analysis. **(D)** Correlation of the 23 prognostic crg. **(E)** A PPI network indicating the interactions among the 23 prognostic CRGs.

**Figure 4:**
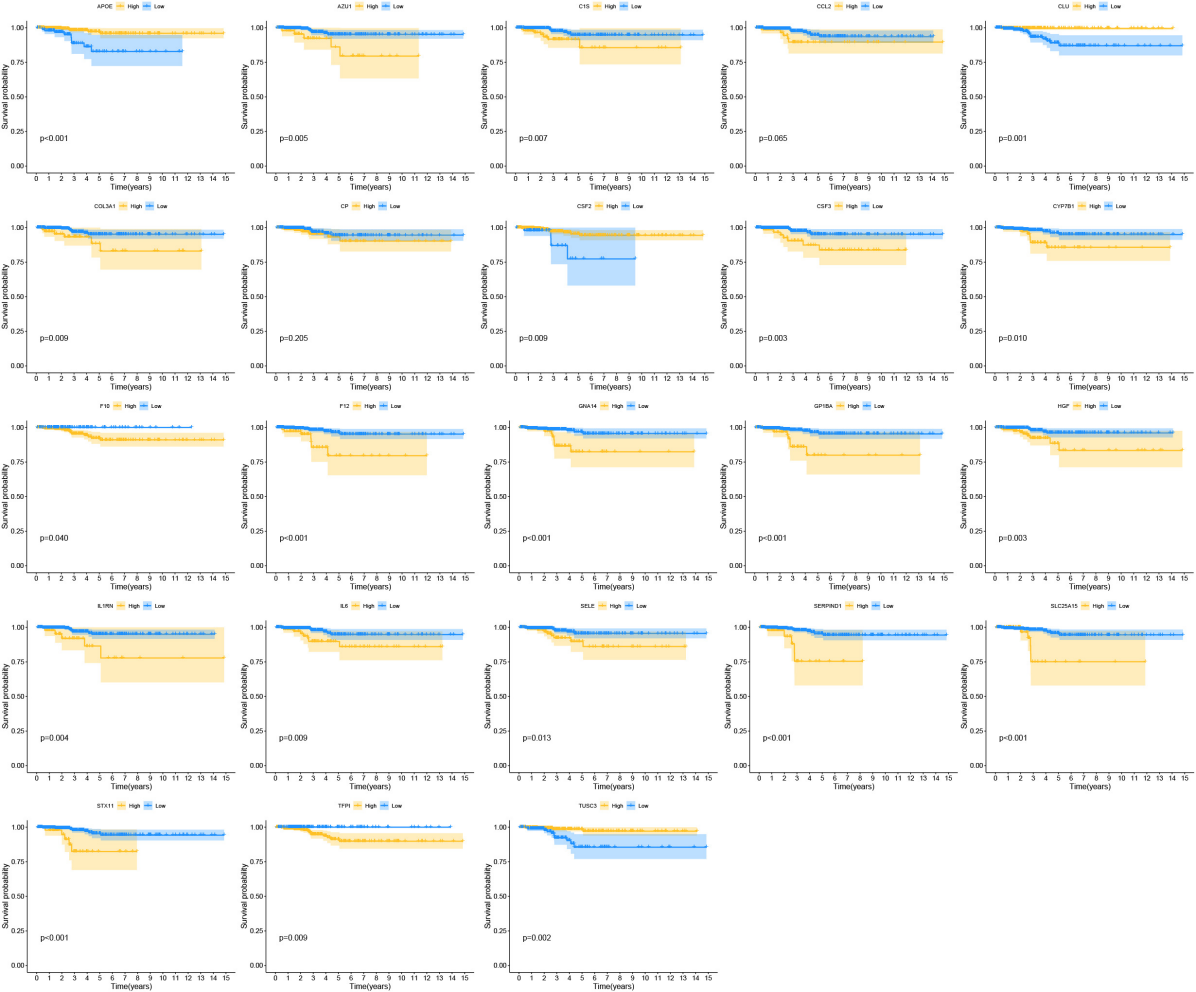
The Kaplan-Meier survival curves of 23 prognostic CRGs in THCA.

By comparing gene expression levels in tumor and normal tissue samples, we identified 1374 differentially expressed genes. The heatmap displaying differentially expressed genes is shown in **Figure 3A**. For deeper analysis, we obtained 377 CRGs from the Genecards website (https://www.genecards.org), and 64 of these CRGs were differentially expressed between THCA tissues and normal controls as shown in the Venn plot (**Figure 3B**). We used univariate COX regression analysis to assess the relationship between the 64 CRGs and PFI, identifying 23 prognostic CRGs (**Figure 3C**). The correlations of these genes are shown in **Figure 3D**. Additionally, we constructed a PPI network of the 23 prognostic CRGs (**Figure 3E**). The Kaplan-Meier survival curves of 23 prognostic CRGs in THCA are shown in **Figure 4**.

### Identification of coagulation-related subtypes

**Figure 5:**
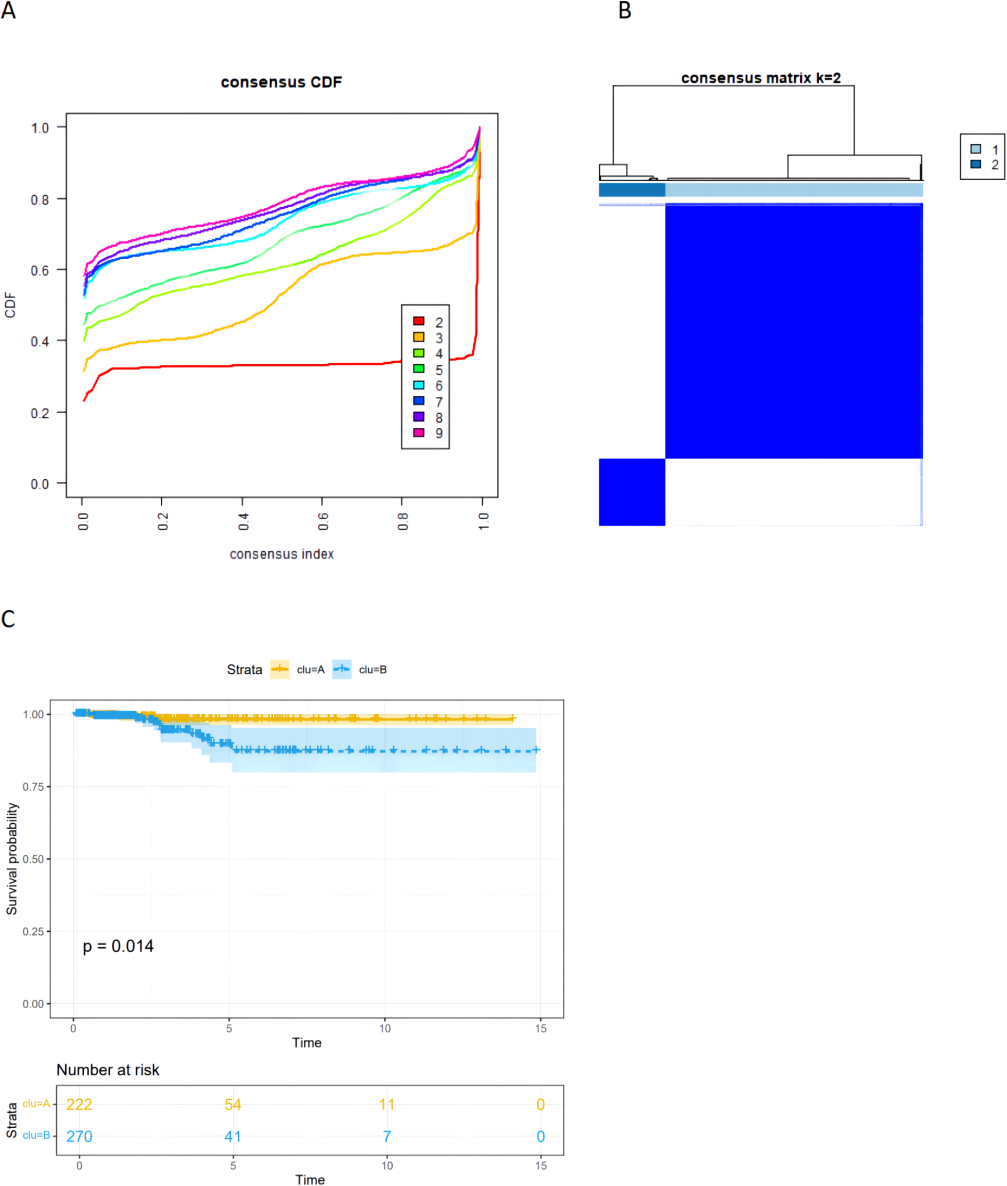
**(A)** The CDF curves of the consensus cluster. **(B)** The heatmap of consensus matrices for TCGA-THCA patients(k=2). **(C)** The Kaplan–Meier (K–M) survival curves for TCGA-thca patients, which were stratified by the coagulation-related subtypes.

We obtained transcriptomic data and corresponding clinical characteristics of patients from the TCGA-THCA cohort. Based on the 23 prognostic CRGs, we classified THCA patients into two subtypes using the unsupervised clustering method, including coagulation-related cluster 1 and cluster 2 (**Figures 5A, B**). We conducted K-M survival analysis on the TCGA-THCA cohort, and the results indicated that cluster 1 had a better prognosis than cluster 2 (p=0.014) (**Figure 5C**).

### Prognostic model construction and validation

**Figure 6:**
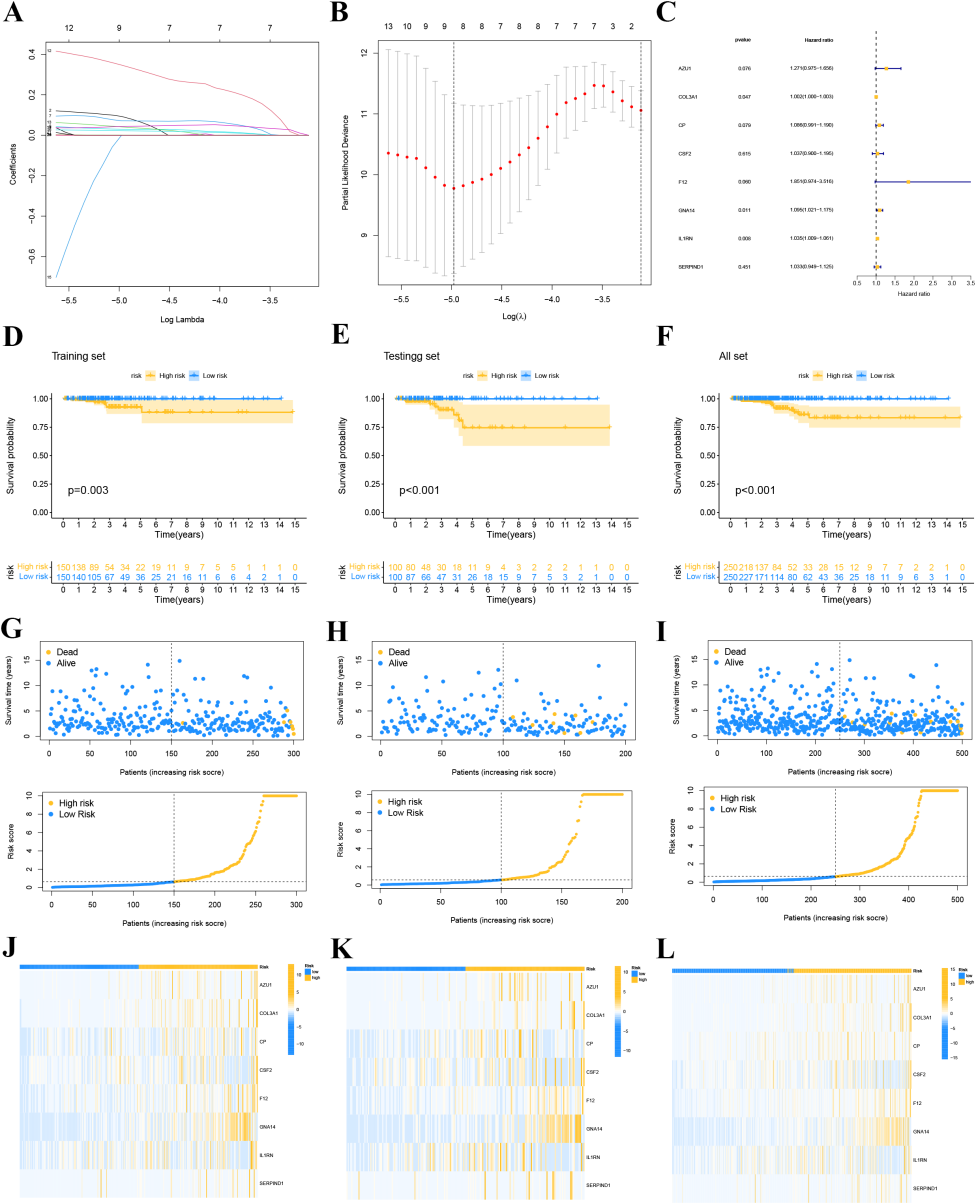
LASSO regression model. **(A)** Coefficient screening was performed based to LASSO analysis. **(B)** Parameters were adjusted by ten cross-validation. **(C)** stepwise multivariate Cox regression analysis. **(D-F)** Survival curve for high and low risk groups in the training set **(D)**, the validation set **(E)** and whole set **(F)**. **(G-I)** Distribution of risk scores and survival status of each THCA patient in the training set **(G)**, validation set **(H)** and whole set **(I)**. **(J-L)** Expression levels of the gene of each THCA patient in the training set **(J)**, validation se t **(K)** and whole set **(L)**.

We divided the patients in the TCGA-THCA dataset into training and validation sets in a 6:4 ratio. In the training cohort of TCGA-THCA, we conducted LASSO regression analysis (**Figures 6A,B**), screening out 8 key genes (AZU1, COL3A1, CP, CSF2, F12, GNA14, IL1RN, and SERPIND1). Using stepwise multivariate Cox regression analysis, we determined the coefficients associated with each gene (**Figure 6C**): Riskscore = 0.23947 * AZU1 + 0.00155 * COL3A1 + 0.08232 * CP + 0.03633 * CSF2 + 0.61566 * F12 + 0.09073 * GNA14 + 0.03401 * IL1RN + 0.03270 * SERPIND1.

We classified THCA patients in the training and validation sets into high-risk and low-risk groups based on the median risk score. **Figures 6G–I** provide detailed information on the risk score distribution and survival status of each THCA patient. Kaplan-Meier survival analysis curves were drawn to compare PFI differences between high- and low-risk groups in the training, validation, and overall sets. The results indicated that PFI was shorter in high-risk patients compared to low-risk patients (all P<0.05, **Figures 6D–F**), suggesting that the prognostic model has clinical utility in predicting THCA prognosis. Our study found that AZU1, COL3A1, CP, CSF2, F12, GNA14, IL1RN, and SERPIND1 were overexpressed in the high-risk group (**Figures 6J–L**).

### Validation of the 8-Gene signature

**Figure 7:**
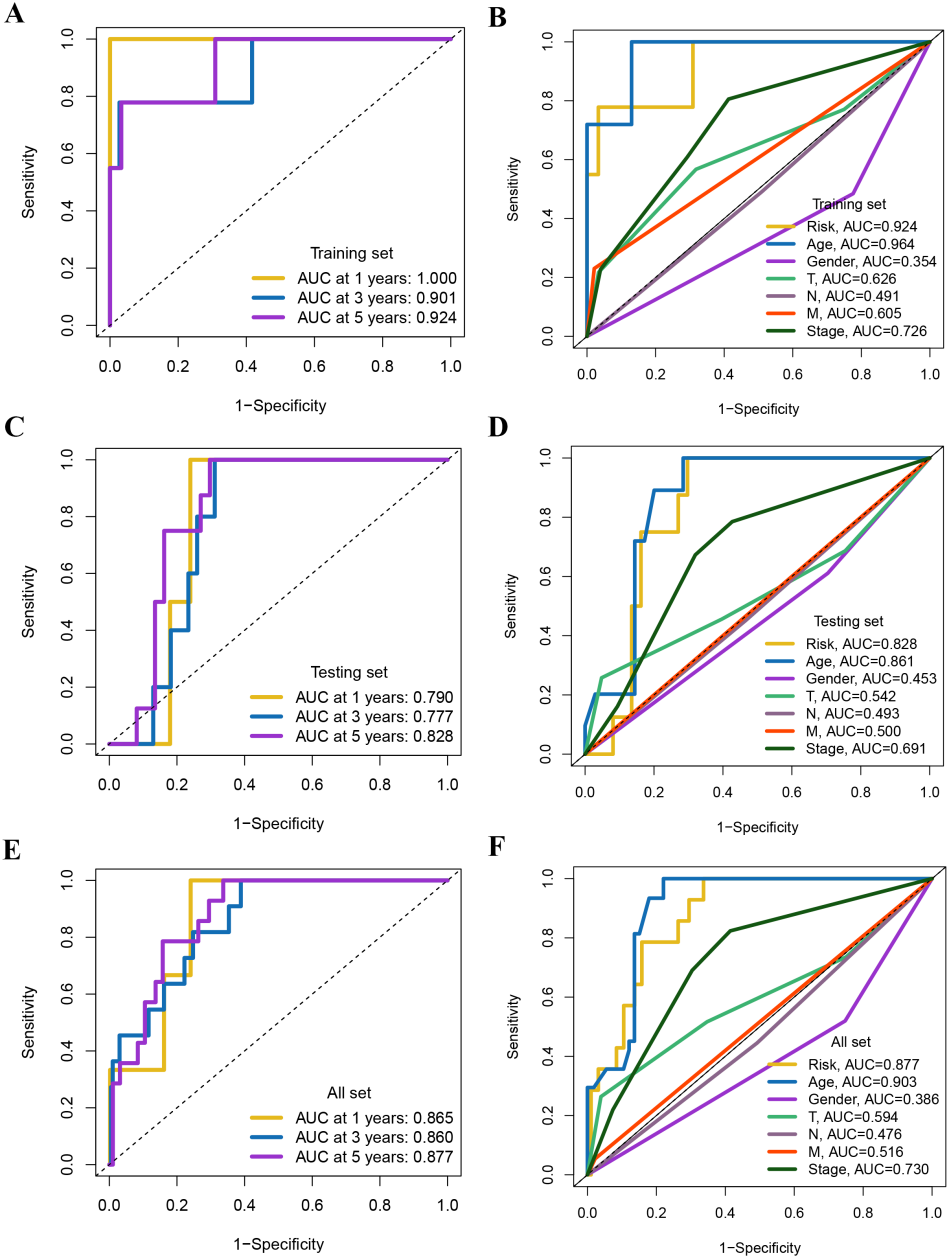
**(A, C, E)** ROC curves for the training set, validation set, and total set based on risk score versus survival state. **(B, D, F)** ROC curves of risk score and each clinical feature for predicting 5-year PFI in the training set, the validation set and whole set.

The ROC curves further showcased the excellent performance of the prognostic model in predicting PFI. We found that the AUC for the prognostic features in the training set reached 1.000, 0.901, and 0.924 at 1, 3, and 5 years, respectively. Likewise, in the test set, the AUC were 0.790, 0.777, and 0.828, respectively. For the entire cohort, the AUC reached 0.865, 0.860, and 0.877, respectively (**Figures 7A, C, E**). **Figures 7B, D, F** illustrate the ROC curves for the risk score and different clinical characteristics predicting 5-year PFI.

**Figure 8:**
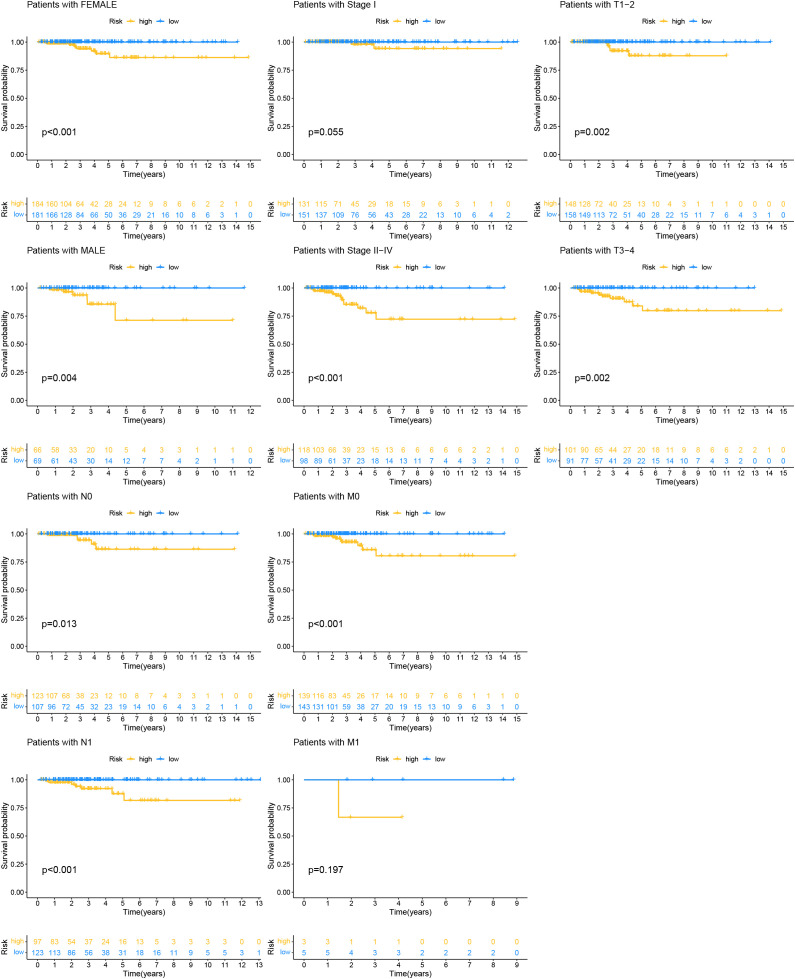
Kaplan-Meier survival analysis based on the grouping of each clinical feature and risk score.

To assess the applicability of the model for patients with various clinical characteristics, we compared survival differences between different risk score groups within each subgroup (**Figure 8**). We found that the prognostic model exhibited good performance in predicting PFI across subgroups including male, female, N0, N1, Stage II-IV, M0, T1-2, and T3-4 (**Figure 8**).

**Figure 9:**
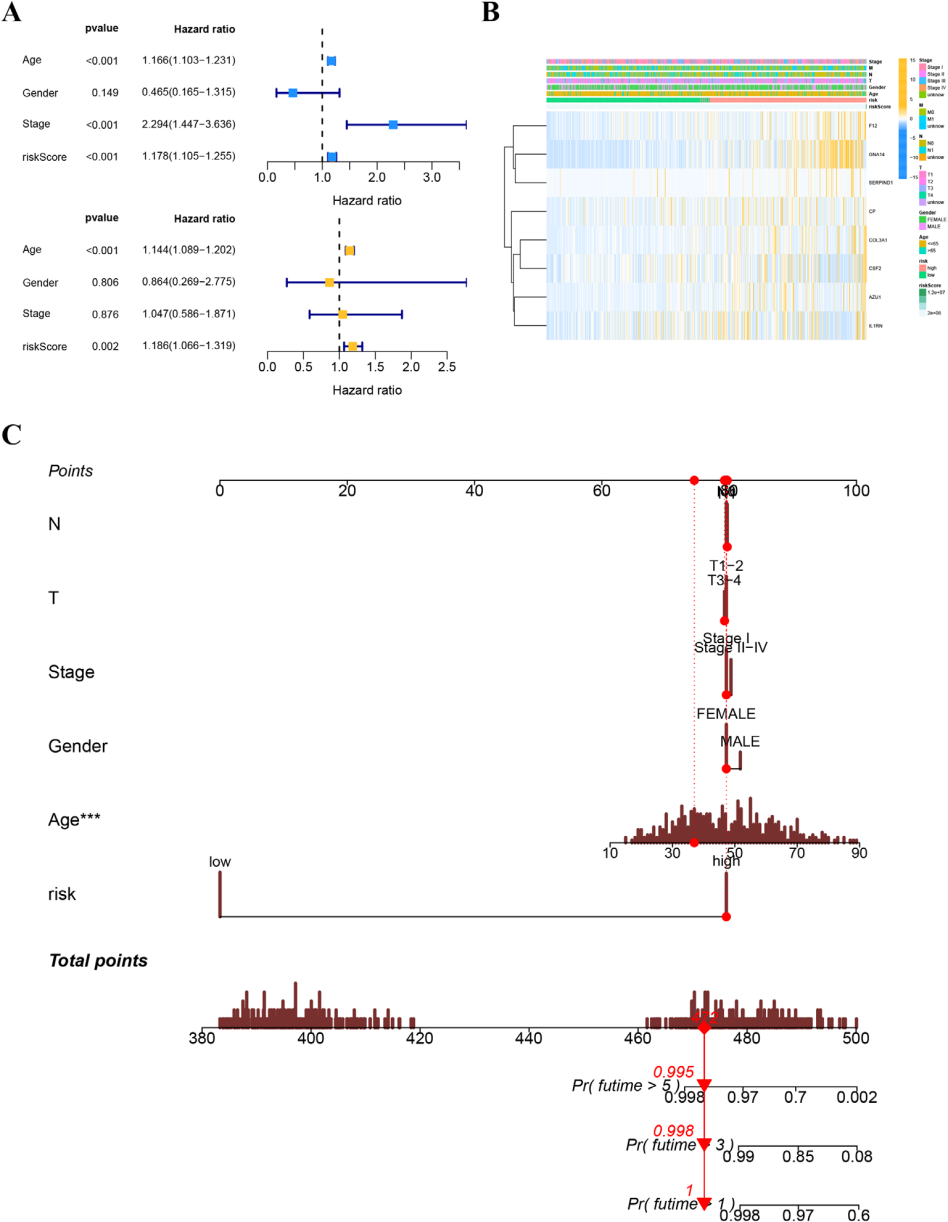
**(A)** Univariate and multivariate Cox analyses for the risk score and other clinical features in TCGA cohort. **(B)** Distribution of clinical features and gene expression in the high-and low-risk groups. **(C)** The nomogram for predicting the survival probability of THCA patients.

**Figure 10:**
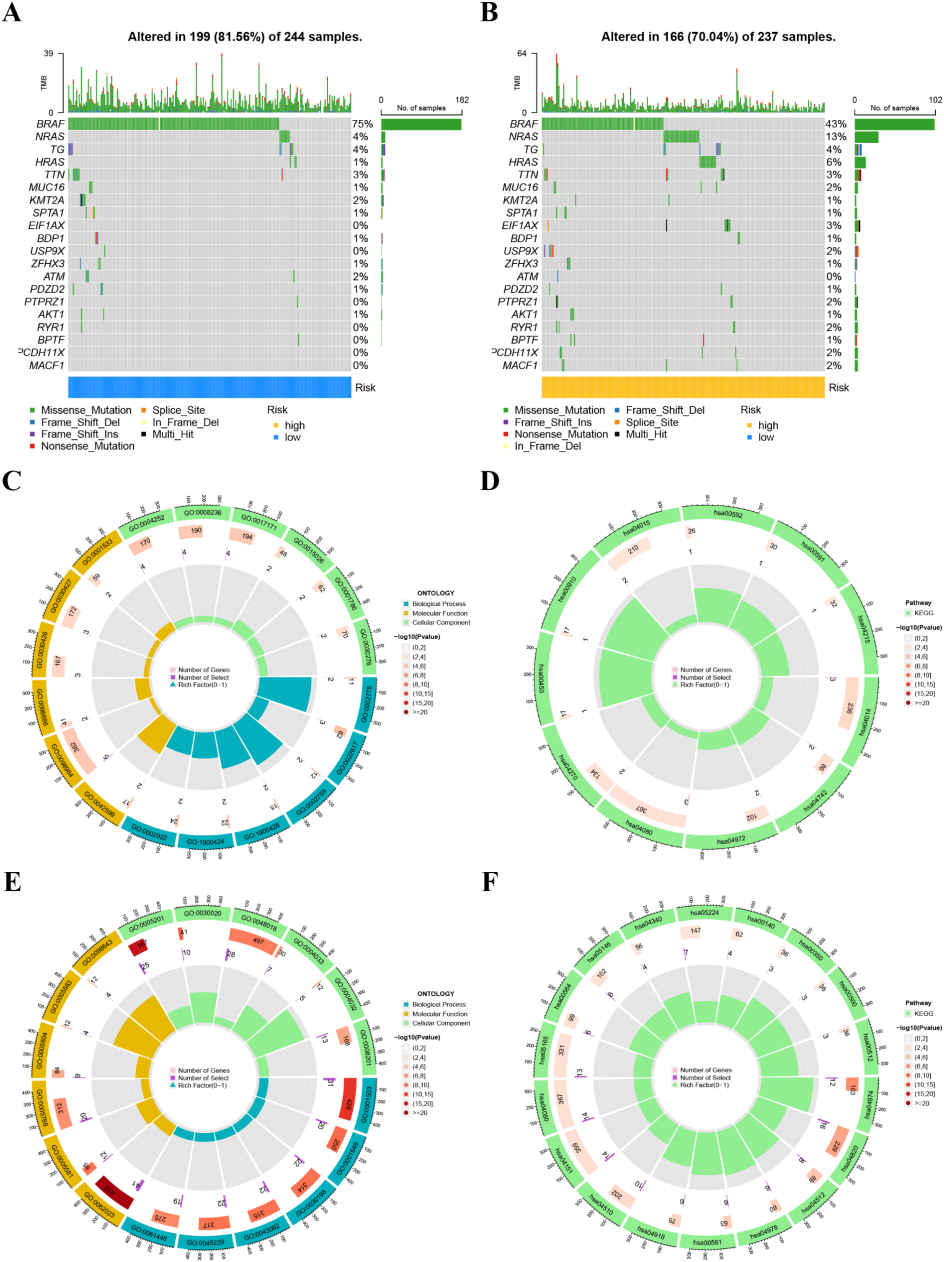
**(A, B)** Tumor mutation profile in the high-risk **(B)** and low-risk **(A)** groups. **(C, D)** Gene Ontology (GO) **(C)** and Kyoto Encyclopedia of Genes and Genomes (KEGG) pathways **(D)** enrichment analysis in the low-risk group. **(E, F)** Gene Ontology (GO) **(E)** and Kyoto Encyclopedia of Genes and Genomes (KEGG) pathways **(F)** enrichment analysis in the high-risk group.

A heatmap illustrated the distribution of clinical characteristics and gene expression in the high-risk and low-risk groups (**Figure 9B**). To assess the independence of the risk score as a prognostic indicator for THCA, univariate and multivariate Cox regression analyses were conducted in the TCGA-THCA cohort. The analyses consistently affirmed the risk score’s status as an independent prognostic predictor (all p < 0.05) (**Figure 9A**). We created a nomogram that integrates the risk score and clinical characteristics to enhance the clinical application of our results (**Figure 9C**). **Figures 10A, B** depict the gene mutation occurrences in the low-risk and high-risk groups, respectively. In the low-risk group, the somatic mutation frequency rankings were BRAF (75%), NRAS (4%), TG (4%), with mutation frequencies of other genes below 4%. In the high-risk group, the somatic mutation frequency rankings were BRAF (43%), NRAS (13%), HRAS (6%), TG (4%), with mutation frequencies of other genes below 4%. It is noteworthy that the primary type of somatic mutation in both high- and low-risk groups was missense mutation, indicating that missense mutations may play a crucial role in the common mechanisms of tumorigenesis across different risk levels.

### Functional, immune infiltration analyses and drug sensitivity analysis in different risk groups

We examined the biological functions and pathways of the high-risk and low-risk groups using GO and KEGG pathway analyses. GO analysis indicated that the Biological process, cellular component, and Molecular Function affected in the low-risk group mainly included antimicrobial peptide production, extracellular matrix disassembly, lamellar body, neuron to neuron synapse, hippocampal mossy fiber to CA3 synapse, serine-type endopeptidase activity, and serine-type peptidase activity (**Figure 10C**). KEGG analysis revealed that the significantly enriched pathways in the low-risk group were the Ras signaling pathway, Taste transduction, Pancreatic secretion, and Neuroactive ligand-receptor interaction (**Figure 10D**). GO analysis indicated that the Biological process, cellular component, and Molecular Function affected in the high-risk group mainly included ossification, osteoblast differentiation, collagen-containing extracellular matrix, collagen trimer, extracellular matrix structural constituent, and extracellular matrix structural constituent conferring tensile strength (**Figure 10E**). KEGG analysis revealed that the significantly enriched pathways in the high-risk group were Protein digestion and absorption, Cytoskeleton in muscle cells, and ECM-receptor interaction (**Figure 10F**).

**Figure 11:**
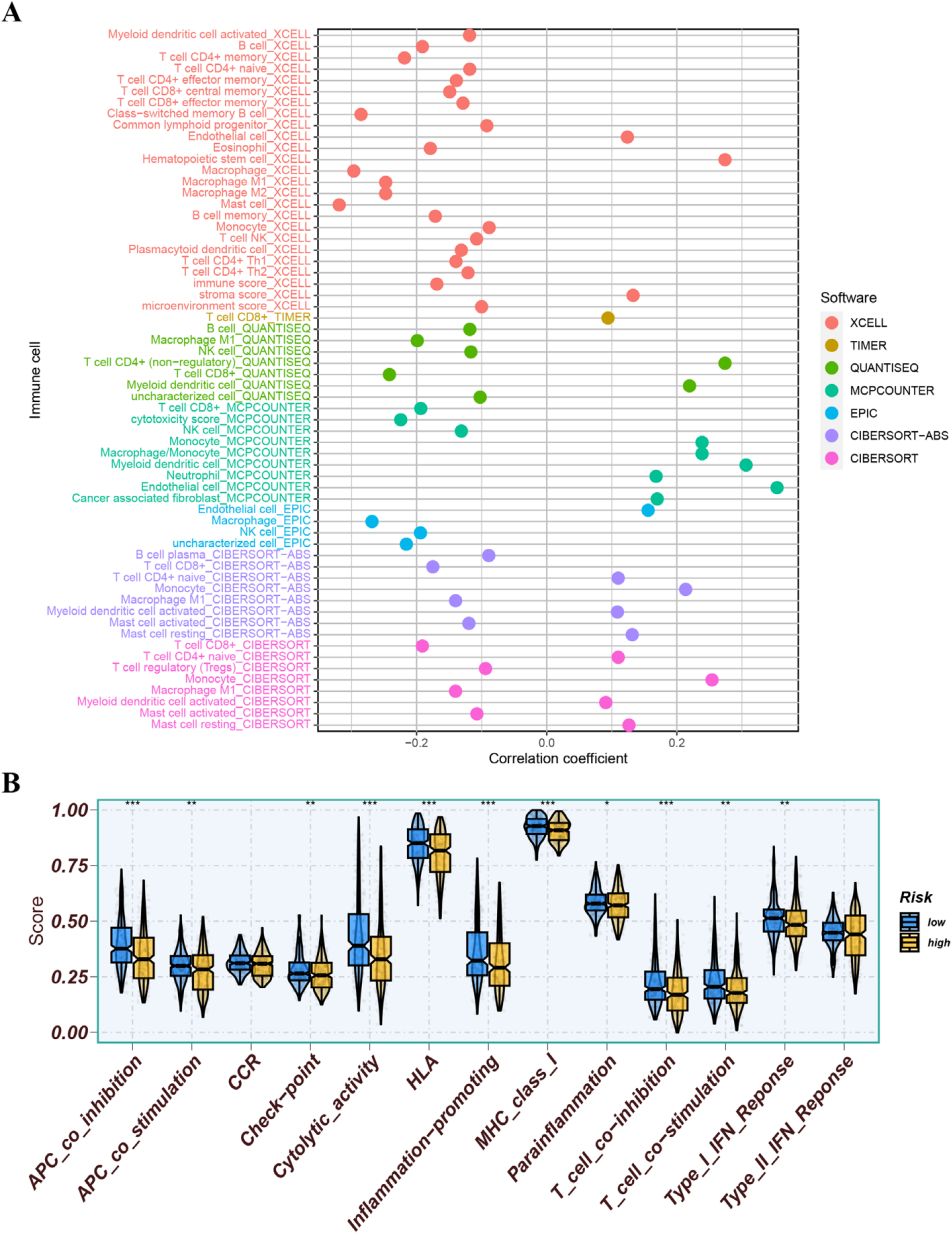
**(A)** Correlation analysis of risk score with immune cells. **(B)** The immune function of different risk groups. ns indicates P>0.5, * indicates P<0.05, ** indicates P<0.01, *** indicates P<0.001.

**Figure 12:**
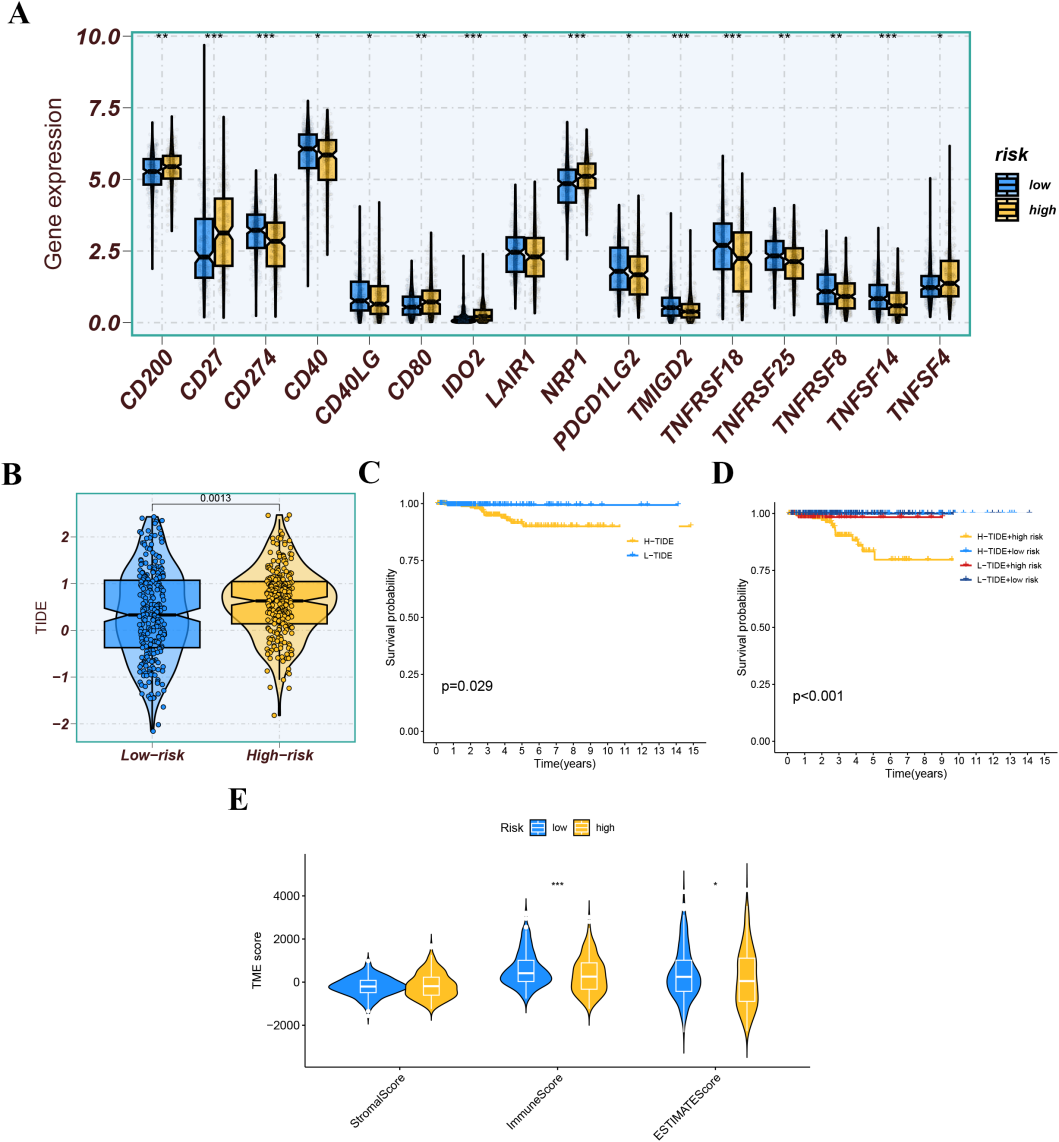
**(A)** Expression levels of immune checkpoint molecules between different risk groups. **(B)** The tumor immune dysfunction and exclusion (TIDE) score for each group. **(C, D)** Kaplan-Meier survival analysis based on the TIDE score and risk score. **(E)** TME score (StromalScore, ImmuneScore, and EstimateScore) across different risk groups. ns indicates P>0.5, * indicates P<0.05, ** indicates P<0.01, *** indicates P<0.001.

We further analyzed the differences in immune infiltration levels between the high-risk and low-risk groups. The results indicated that the risk score was negatively correlated with the infiltration levels of macrophage M1 and M2 subtypes, CD8+ T cells, and NK cells, with correlation coefficients all below -0.2 (**Figure 11A**), suggesting that the role of these cells may weaken as the risk score increases. The immune function analysis of different risk groups is depicted in **Figure 11B**. The differential expression analysis of immune checkpoints between different risk groups showed that immune checkpoints significantly expressed in the low-risk group compared to the high-risk group included CD274, TMIGD2, and TNFRSF18, while immune checkpoints significantly expressed in the high-risk group compared to the low-risk group included CD27, IDO2, and NRP1 (**Figure 12A**). We also calculated the Tumor Immune Dysfunction and Exclusion (TIDE) score for each group and compared them. The results indicated that the high-risk group had higher TIDE scores, and higher TIDE scores were associated with poorer prognosis. The group with high TIDE scores and high-risk scores had a relatively worse prognosis compared to other groups (**Figures 12B–D**). TME scores also suggested better prognosis for the low-risk group (**Figure 12E**).

**Figure 13:**
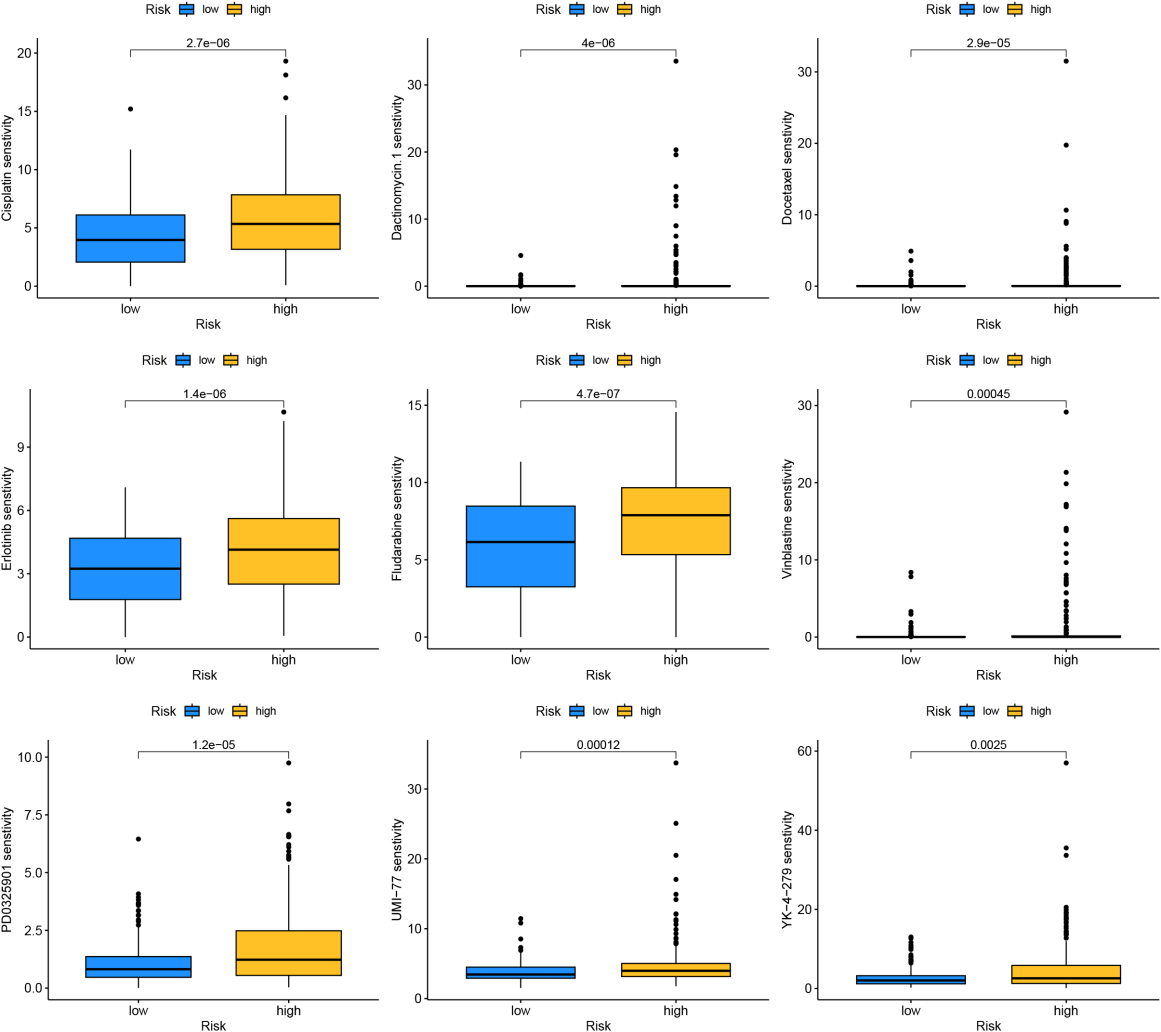
Drug sensitivity analysis for the different risk groups.

Drug sensitivity analysis results indicated that drugs such as Cisplatin, Dactinomycin.1, Docetaxel, Erlotinib, and Fludarabine had higher sensitivity in the high-risk group, implying they might have greater potential in the treatment of patients in the high-risk group (**Figure 13**).

### Validation of the genes expression

**Figure 14:**
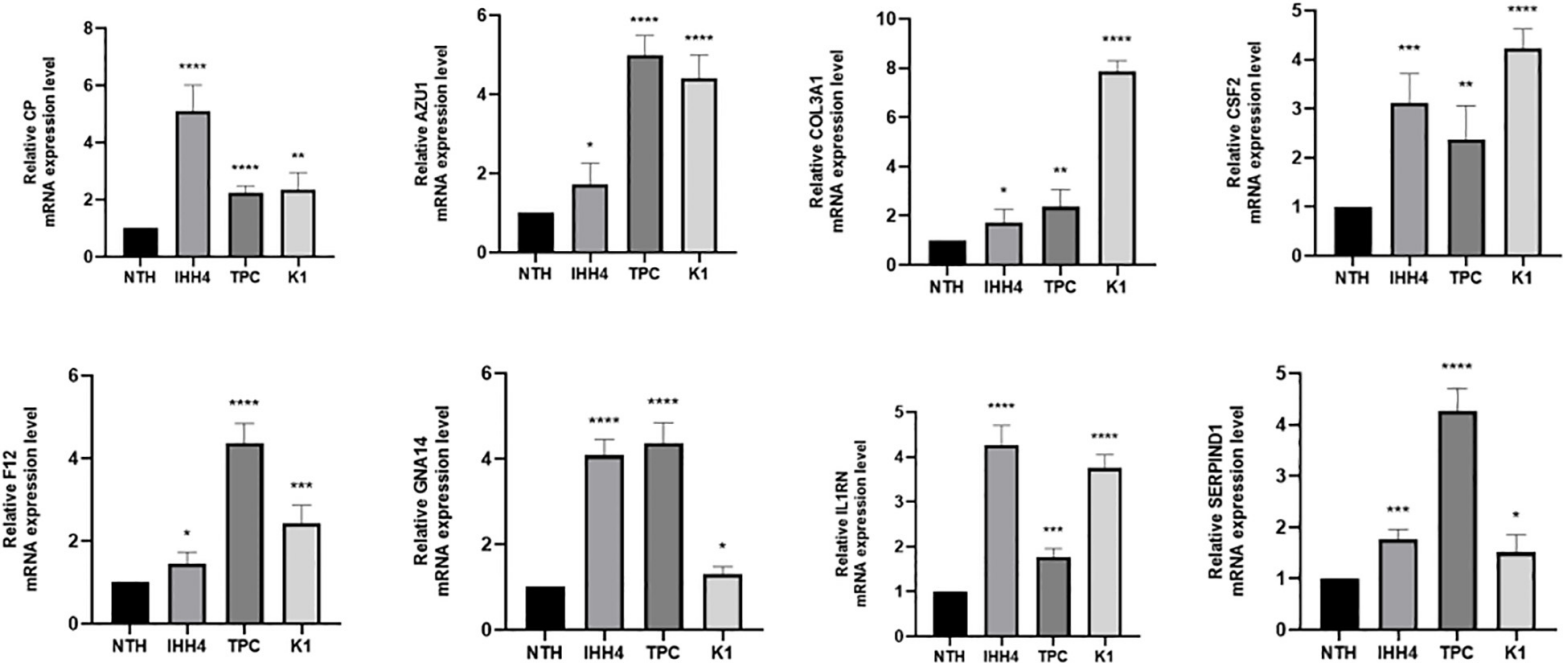
Expression of 8 genes in IHH 4, KTC-1, TPC-1 human papillary thyroid carcinoma cell line and human normal thyroid cell line Nthy ori-3-1. ns indicates P>0.5, * indicates P<0.05, ** indicates P<0.01, *** indicates P<0.001.

The expression levels of 10 SHMRGs were detected in human PTC cell lines (IHH4, KTC-1, TPC-1) and human normal thyroid cell line (Nthy ori-3-1) using qPCR technology (**Figure 14**).

## Discussion

THCA accounts for 3% of all cancers. While it is mostly regarded as an indolent disease, its recurrence rate remains at 10%-15% (4). Generally, cancer is associated with an imbalance in the hemostatic system, but the pathogenesis of cancer-related coagulation disorders is complex (30). However, there is limited research on the relationship between THCA and coagulation. This study aims to clarify the correlation between coagulation and prognosis in THCA patients.

Several studies have indicated that PTC patients with LLNM have a higher risk of recurrence compared to those without LLNM (31, 32). Generally, prophylactic central lymph node dissection is deemed necessary. However, prophylactic dissection of negative lateral lymph nodes is not recommended because it may lead to numerous surgical complications. Therefore, it is important to determine the presence of LLNM preoperatively. This study analyzed the influence of coagulation markers (PT, PTA, INR, APTT, fibrinogen, TT, and D-dimer) on LLNM. The ROC curve showed that D-dimer has a good predictive performance for LLNM. Multivariate analysis indicated that extrathyroidal extension, multifocality, and D-dimer >0.065mg/l are independent predictors of LLNM. The effects of extrathyroidal extension and multifocality on LLNM have been confirmed in numerous studies (33, 34). The research has shown that elevated D-dimer is significantly associated with cancer recurrence, metastasis, and worse survival outcomes (35). This study suggests that D-dimer >0.065mg/l is significantly associated with LLNM in PTC.

In this study, we divided THCA patients into two distinct subtypes based on the expression of prognostically valuable CRGs. K-M survival analysis indicated that cluster1 had better outcomes than cluster2 (p=0.014), offering a reference for refining the classification and management of THCA patients in clinical practice. Subsequently, we used LASSO regression to screen 8 genes (AZU1, COL3A1, CP, CSF2, F12, GNA14, IL1RN, and SERPIND1) to construct the prognostic model. ROC results from both the training and validation sets suggested that the constructed model had high predictive efficacy, and the high-risk group had poorer outcomes. We further used univariate and multivariate Cox regression analyses to validate the effectiveness of the prognostic model. We also developed a nomogram integrating risk scores and clinical features to facilitate the clinical application of our research findings.

The tumor immune microenvironment (TME) plays an important role in tumor development. Recently, a pan-cancer analysis of the human tumor coagulome revealed that coagulation links to the TME (36). In our study results, the high-risk group had lower infiltration levels of macrophage M1 and M2 subtypes, CD8+ T cells, and NK cells, which may be related to poorer prognosis. Macrophages may combat cancer progression by directly engulfing cancer cells or activating antitumor immune responses, and CD8+ T cells and NK cells also have strong antitumor functions (37). To explore the biological functional differences between high and low-risk groups, we conducted GO and KEGG analyses. The results indicated significant differences in biological functions between the two groups, but the underlying mechanisms remain to be clarified. Finally, we investigated the intrinsic connection between drug sensitivity and risk groups by correlating THCA patients’ prognostic risk scores with the half-maximal inhibitory concentration (IC50) values of chemotherapeutic drugs.

Research has indicated that these genes are involved in cancer development. In gastric cancer, AZU1 was upregulated (38). COL3A1 could be an oncogene and promote drug resistance in lung cancer (39). The product expressed by CP, cancer procoagulant, is a biomarker for cancer. Elevated CP activity has been detected in pancreatic, breast, lung, digestive system, and urinary system cancers (40). In breast cancer, CSF2 could activate the Stat3 pathway in CAA via paracrine or autocrine mechanisms, leading to increased expression and secretion of CXCL3. CXCL3 binds to CXCR2, a receptor on breast cancer cells, and activates FAK, which promotes a mesenchymal phenotype, invasion, and metastasis of breast cancer cells (41) F12 expression might affect the OS of PTC patients by regulating metabolic pathways (42). GNA14 might accelerate colorectal cancer cell proliferation and malignant tumor progression through ERK and β-catenin pathways (43). IL1RN was a good prognostic and diagnostic biomarker for PTC and might promote thyroid cancer progression through immune-related pathways (44). SERPIND1 promoted the proliferation, migration, invasion, G1-to-S phase transition, and epithelial-mesenchymal transition of ovarian cancer cells and inhibited their apoptosis by promoting phosphorylation in the phosphoinositide 3-kinase/protein kinase B (PI3K/AKT) pathway (36). We also validated the high expression of these 8 genes in PTC by qRT-PCR.

There are some limitations in this study. Firstly, the TCGA-THCA cohort has a limited number of cases, and more datasets are needed to validate these findings. Otherwise, the selected genes were only validated by qRT-PCR in PTC, lacking comprehensive experimental validation. Moreover, although we found that the coagulation pathway affects the prognosis and immune microenvironment of THCA patients, its underlying mechanisms need further investigation.

## Conclusion

Our study demonstrated that coagulation is related to immune infiltration and prognosis in THCA. Our study suggested the possibility of D-dimer predicting LLNM. Subsequently, we used the TCGA cohort to construct a new coagulation-related THCA risk scoring model for prognosis assessment and risk stratification in THCA patients. This risk model could provide a robust prognostic tool and promote clinical guidance for THCA patients. We also constructed a nomogram combining the model and clinical features to predict the 3-, 5-, and 7-year survival probabilities of patients. We analyzed and compared high- and low-risk groups regarding immune infiltration, somatic mutations, and other aspects, offering some guidance for treatment strategy selection. In summary, our research will aid in understanding the role and significance of the coagulation in THCA.

## Data Availability

The original contributions presented in the study are included in the article/**Supplementary Materials**. Further inquiries can be directed to the corresponding author/s.
